# G-CSF-induced Aortitis in an elderly woman successfully managed with prednisolone: a case report and literature review

**DOI:** 10.1093/omcr/omaf144

**Published:** 2025-08-25

**Authors:** Taeko Kurosawa, Masahiro Ohara, Ayako Nakame, Yuki Ichinose, Akihiro Fujimoto, Asami Nukui, Kei Yamaguchi, Aya Asano, Hiroko Shimada, Hideki Yokogawa, Kazuo Matsuura, Hiroshi Ishiguro, Takahiro Hasebe, Akihiko Osaki, Toshiaki Saeki

**Affiliations:** Department of Breast Oncology, Saitama Medical University International Medical Center, 1397-1 Yamane, Hidaka, Saitama 350-1298, Japan; Department of Breast Oncology, Saitama Medical University International Medical Center, 1397-1 Yamane, Hidaka, Saitama 350-1298, Japan; Department of Breast Oncology, Saitama Medical University International Medical Center, 1397-1 Yamane, Hidaka, Saitama 350-1298, Japan; Department of Breast Oncology, Saitama Medical University International Medical Center, 1397-1 Yamane, Hidaka, Saitama 350-1298, Japan; Department of Breast Oncology, Saitama Medical University International Medical Center, 1397-1 Yamane, Hidaka, Saitama 350-1298, Japan; Department of Breast Oncology, Saitama Medical University International Medical Center, 1397-1 Yamane, Hidaka, Saitama 350-1298, Japan; Department of Breast Oncology, Saitama Medical University International Medical Center, 1397-1 Yamane, Hidaka, Saitama 350-1298, Japan; Department of Breast Oncology, Saitama Medical University Hospital, 38 Morohongo, Moroyama-machi,Iruma-gun, Saitama 350-0495, Japan; Department of Breast Oncology, Saitama Medical University International Medical Center, 1397-1 Yamane, Hidaka, Saitama 350-1298, Japan; Department of Breast Oncology, Saitama Medical University International Medical Center, 1397-1 Yamane, Hidaka, Saitama 350-1298, Japan; Department of Breast Oncology, Saitama Medical University International Medical Center, 1397-1 Yamane, Hidaka, Saitama 350-1298, Japan; Department of Breast Oncology, Saitama Medical University International Medical Center, 1397-1 Yamane, Hidaka, Saitama 350-1298, Japan; Department of Breast Oncology, Saitama Medical University International Medical Center, 1397-1 Yamane, Hidaka, Saitama 350-1298, Japan; Department of Breast Oncology, Saitama Medical University International Medical Center, 1397-1 Yamane, Hidaka, Saitama 350-1298, Japan; Department of Breast Oncology, Saitama Medical University International Medical Center, 1397-1 Yamane, Hidaka, Saitama 350-1298, Japan

**Keywords:** granulocyte-colony stimulating factor, Aortitis, breast cancer, Pegfilgrastim, Filgrastim

## Abstract

Cancer incidence among old is increasing. Since age is important risk factor for febrile neutropenia (FN), use of granulocyte-colony stimulating factor (G-CSF) and its complication is clinically important. A 72-year old woman has completed definitive surgery for left breast cancer and was started on postoperative chemotherapy. After 12 doses of paclitaxel, she received 1st cycle of epirubicin and cyclophosphamide (day 1), and pegfilgrastim, a pegylated G-CSF to decrease the risk of FN (day 2). On day 13, she was admitted due to persistent fever since day 9. Laboratory tests revealed elevated neutrophil counts and C-reactive protein. Despite empirical antibiotics, her fever persisted and severe back pain developed (day 15). Contrast-enhanced computed tomography revealed wall thickening and increased density around the aortic arch and brachiocephalic artery. Diagnosis of pegfilgrastim-induced vasculitis was made after excluding autoimmune vasculitis. Prednisolone (60 mg/day) was administered and the fever and back pain subsided the following day.

## Introduction

Granulocyte colony-stimulating factor (G-CSF) is commonly used for the primary or secondary prevention of chemotherapy-related febrile neutropenia (FN). The typical side effects of G-CSF include musculoskeletal pain, headache, injection site reactions, and liver dysfunction [1–3]. Pegfilgrastim is a commonly used pegylated G-CSF preparation. Aortitis is a rare complication of G-CSF, and its precise incidence is unknown.

We report a rare case of G-CSF-induced aortitis in an older woman that was successfully managed with prednisolone (PSL).

## Case report

Based on pathological examination following mastectomy with axillary lymph node dissection, a 72-year-old woman was diagnosed with left-sided breast cancer (T2N1M0, Stage IIB) that was categorized as estrogen receptor, progesterone receptor, and human epidermal growth factor receptor type-2 negative invasive ductal breast carcinoma.

Twelve doses of weekly paclitaxel and four cycles of epirubicin cyclophosphamide (EC) were scheduled as postoperative adjuvant therapy. Blood tests from the first cycle (day 1) of EC showed a mild decrease in white blood cell (WBC)(3150/μl) and neutrophil counts (2053/μl), normal platelet counts (294 000/μl), a mild decrease in albumin (4.1 g/dl), and a mild increase in C-reactive protein (CRP) (0.101 mg/dl); the first EC cycle was administered as scheduled. Following the first cycle of EC (day 1), pegfilgrastim was administered via an on-body injector 27 h later (day 2). Due to a persistent fever (body temperature ≥ 39.0°C) starting on day 9, the patient was admitted to the hospital on day 13 for thorough examination and immediate treatment.

The patient was conscious, physically well, and showed no infectious manifestations. Laboratory tests at admission revealed high WBC (18 240/μl), neutrophil (15 887/μl), and platelet counts (328 000/μl), a low level of albumin (2.5 g/dl), and a high C-reactive protein (CRP) level (28.035 mg/dl) without any other abnormalities.

The patient tested negative for influenza and coronavirus disease 2019 antigens. Non-contrast-enhanced computed tomography (CT) during hospitalization did not reveal the cause of the fever. After various culture tests were conducted, antibiotics (sulbactam/ampicillin) were administered due to suspicions of infection. However, blood culture, β-D glucan, and urine culture tests yielded negative results.

**Figure 1:**
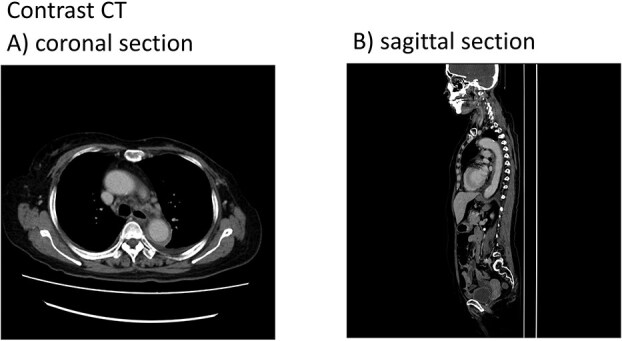
Contrast computed tomography findings: A) coronal section and B) sagittal section. Contrast computed tomography revealed wall thickening and increased density around the aortic arch and brachiocephalic artery.

On day 15 (hospitalization day 3), the patient developed persistent fever, back pain, tachypnea, and respiratory discomfort that prompted contrast-enhanced CT that revealed wall thickening and increased density around the aortic arch and brachiocephalic artery along with bilateral pleural effusions (predominantly on the left side) (Fig. 1).

Therefore, vasculitis was suspected, and PSL was initiated at a dose of 1.0 mg/kg/day (60 mg/day), in accordance with clinical guideline [4], in combination with prophylactic sulfamethoxazole-trimethoprim medication to prevent Pneumocystis pneumonia infection. Concurrently submitted tests for Anti–membrane attack complex (MAC) antibodies, antinuclear antibodies, myeloperoxidase-anti-neutrophil cytoplasmic antibodies (MPO-ANCA), and proteinase-3-anti-neutrophil cytoplasmic antibodies (PR3-ANCA) were performed to eliminate autoimmune vasculitis; these tests yielded negative results, thereby increasing the likelihood of vasculitis due to pegfilgrastim.

**Figure 2:**
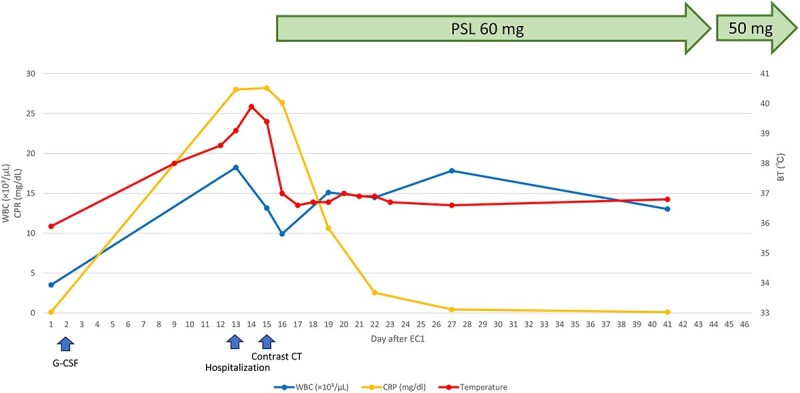
Changes in body temperature, WBC count, and CRP level after the administration of G-CSF. The fever resolved following steroid administration, and both the WBC count and CRP level decreased. The treatment regimen commenced with PSL at 60 mg and continued for 30 days, after which it was gradually tapered down to PSL 50 mg. WBC; white blood cell, CRP; C-reactive protein, BT; body temperature, G-CSF; granulocyte-colony stimulating factor, EC; epirubicin cyclophosphamide, CT; computed tomography, PSL; prednisolone.

Following the initiation of steroid therapy, the patient’s temperature subsided to 36.7°C on day 17 (day 4 of hospitalization). Additionally, the WBC count and CRP levels improved, and the patient was discharged on day 25 following symptom improvement. On day 44, the PSL dose was tapered to 50 mg and reduced by 5 mg every 2 weeks (Fig. 2).

Due to the patient’s age (72 years) and high risk of FN, it was difficult to continue without G-CSF. Therefore, EC therapy was not resumed, and follow-up for breast cancer was conducted in accordance with the guidelines. PSL was tapered on an outpatient basis. Five months after onset, no symptom relapse was reported.

## Discussion

In various types of carcinomas, G-CSF is widely used to treat or prevent FN induced by cytotoxic chemotherapy. G-CSF promotes neutrophil differentiation and proliferation in the bone marrow. Although generally well-tolerated, G-CSF has been associated with mild side effects, such as muscle pain and fever, and rare but severe complications, such as aortitis, that are believed to result from the increased production of inflammatory cytokines. Aortitis can be classified as either non-infectious or infectious, with the latter primarily linked to autoimmune diseases characterized by increased inflammatory cytokine levels [2]. Although autoimmune disease was a differential diagnosis in this patient, anti-MAC antibody, anti-nuclear antibody, MPO-ANCA, and PR3-ANCA tests yielded negative results, ultimately resulting in a diagnosis of aortitis attributed to G-CSF administration.

A literature search utilizing PubMed and the keywords ‘G-CSF,’ ‘breast cancer,’ and ‘aortitis’ identified 19 reported cases of aortitis associated with G-CSF administration in patients with breast cancer. We also reviewed the references cited in these original articles and ultimately identified 20 cases of G-CSF-associated aortitis, including the present case. We selected cases where the onset, diagnosis, and treatment were sufficiently detailed (Table 1).

**Table 1:** Occurrence of aortitis following G-CSF administration in patients with breast cancer.

Case	Age	G-CSF	Symptoms	Time of onset	Diagnostic method	Site of inflammation	Treatment	Author	Year
1	70	G-CSF	Fever, diarrhea, fainting, and dehydration	Day 9	Contrast CT and PET-CT	Thoracic aorta and brachiocephalic artery	PSL 1 mg/kg	Parodis et al.	2019
2	72	Pegfilgrastim	Fever	Day 5	PET-CT	Thoracic aorta	None	Hoshina et al.	2019
3	43	Pegfilgrastim	Fever	Day 8	Contrast CT	Aortic arch	PSL 0.3 mg/kg	Koyama et al.	2021
4	66	Pegfilgrastim	Fever and left anterior neck pain	Day 11	Contrast CT	Left common carotid artery	None	Nakamura et al.	2020
5	45	Pegfilgrastim	Fever, fatigue, and abdominal discomfort	Day 8	Contrast CT	Aortic arch and ventral aorta	PSL 1 mg/kg	Mukai et al.	2020
6	70	Pegfilgrastim	Fever, right neck pain, and right shoulder pain	Day 8	Contrast CT	Aortic arch, right common carotid artery, brachiocephalic artery, and aortic dissection (StanfordA)	PSL 0.5 mg/kg	Shiraki et al.	2022
7	63	Pegfilgrastim	Fever, loss of appetite, and shortness of breath	Day 8	Contrast CT	Aortic arch, thoracoabdominal aorta, and left subclavian artery	None	Matsumoto et al.	2022
8	40	Lipegfilgrastim	Fever, sore throat, chest pain Recurrence Fatigue, sore throat, and neck pain	Day 3 Day 10	CT, US, and MRI	Carotid artery	Glucocorticoid	Taimen et al.	2017
9	53	Pegfilgrastim	Fever, sore throat, earache, respiratory discomfort, and chest pain	Day 1	CT and MRI	Aorta	Glucocorticoid	Taimen et al.	2016
10	56	Lipegfilgrastim	Fever, neck pain, jaw pain, and fatigue	Day 8	MRI	Carotid artery and thoracic aorta	Glucocorticoid	Taimen et al.	2018
11	70	Lipegfilgrastim	Fever	Day 5	CT	Aorta	None	Taimen et al.	2018
12	62	Pegfilgrastim	Fever	Day 3	CT and PET-CT	Aorta	Glucocorticoid	Taimen et al.	2018
13	52	Filgrastim	Fever and chest pain	Day 6	CT	Aorta	Glucocorticoid	Taimen et al.	2018
14	45	Pegfilgrastim・ Filgrastim (Recurrence)	Fever, muscle pain, chills, and epigastric discomfort	Day 12 Day 10	Contrast CT	Aortic arch and ventral aorta	PSL 0.5 mg/kg	Lee et al.	2015 ~ 2020
15	66	Pegfilgrastim	Fever, muscle pain, chills, and nausea	Day 13	Contrast CT	Aortic arch, ventral aorta, brachiocephalic artery, and left common carotid artery	PSL 0.5 mg/kg	Lee et al.	2015 ~ 2020
16	49	Pegfilgrastim	Fever, muscle pain, and chest discomfort	Day 15	Contrast CT	Aortic arch and left common carotid artery	PSL 0.5 mg/kg	Lee et al.	2015 ~ 2020
17	50	Pegfilgrastim	Fever and muscle pain	Day 12	Contrast CT	Aortic arch, left common carotid artery, brachiocephalic artery, and left subclavian artery	PSL 0.5 mg/kg	Lee et al.	2015 ~ 2020
18	59	Pegfilgrastim	Fever, muscle pain, and chills	Day 17	Contrast CT	Aortic arch, abdominal and thoracic aorta Left common carotid artery, brachiocephalic artery	PSL 0.5 mg/kg	Lee et al.	2015 ~ 2020
19	53	Pegfilgrastim	Fever, muscle pain, chills, and headache	Day 14	Contrast CT	Aortic arch and left common carotid artery	PSL 0.5 mg/kg	Lee et al.	2015 ~ 2020
This case	72	Pegfilgrastim	Fever, back pain, tachypnea, and respiratory discomfort	Day 8	Contrast CT	Aortic arch and brachiocephalic artery	PSL 1.0 mg/kg		2023

G-CSF; granulocyte-colony stimulating factor, CT; computed tomography, PET-CT; Positron Emission Tomography - Computed Tomography, MRI; Magnetic Resonance Imaging.

The patients’ ages ranged from 40 to 72 years (median: 58.4 years), with no significant age variation. The onset of symptoms varied from day 1 to 17 following G-CSF administration, with all patients experiencing persistent fever. Contrast-enhanced CT led to the diagnosis of 16 patients, suggesting the utility of this imaging modality for diagnosing aortitis. Inflammation predominantly involves the aortic arch and is often characterized by continuous capillaries. Pain localized at the vasculitis site is a commonly reported symptom.

Among the various G-CSF preparations, pegfilgrastim was the most commonly used, being administered to 14 patients. Steroids were administered to 15 patients for vasculitis treatment, although three patients experienced symptom improvement even without treatment. Compared with no treatment, steroid administration was associated with a rapid improvement in symptoms. However, the dosage of steroids is determined empirically, thus leading to varying reports and controversies regarding their efficacy. Re-administration of G-CSF was attempted in one patient, resulting in a relapse of vasculitis. Consequently, the re-administration of G-CSF should be approached with caution due to the risk of vasculitis recurrence. In cases where G-CSF administration is contraindicated due to the risk of adverse events like aortitis, alternative strategies should be considered for the management of FN in high-risk patients. These may include the use of prophylactic antibiotics to prevent infections and adjusting chemotherapy regimens to reduce the risk of severe neutropenia. Additionally, in patients with a prior history of G-CSF-induced vasculitis or those at elevated risk of inflammatory complications, the use of shorter-acting G-CSF preparations (e.g. filgrastim) may be considered as a potentially safer alternative, given their lower reported association with aortitis compared to long-acting agents such as pegfilgrastim [5].

Following the diagnosis of aortitis, a retrospective analysis of non-contrast-enhanced CT scans taken on day 13 revealed an increase in the density of fatty tissue surrounding the brachiocephalic artery, suggesting that the onset of aortitis likely occurred at that time. Hence, in cases where high fever and elevated CRP levels persist after G-CSF administration, contrast CT should be considered when evaluating aortitis as a potential differential diagnosis.
